# CXCL10^+ ^T cells and NK cells assist in the recruitment and activation of CXCR3^+ ^and CXCL11^+ ^leukocytes during *Mycobacteria*-enhanced colitis

**DOI:** 10.1186/1471-2172-9-25

**Published:** 2008-06-04

**Authors:** Udai P Singh, Rajesh Singh, Shailesh Singh, Russell K Karls, Frederick D Quinn, Dennis D Taub, James W Lillard

**Affiliations:** 1 Department of Microbiology, Biochemistry, & Immunology, Morehouse School of Medicine, Atlanta, GA, USA; 2 Department of Microbiology & Immunology, University of Louisville, Louisville, KY, USA; 3 Department of Infectious Diseases, College of Veterinary Medicine, University of Georgia, Athens, GA, USA; 4 Laboratory of Immunology, National Institute of Aging, Gerontology Research Center, Baltimore, MD, USA

## Abstract

**Background:**

The role of *Mycobacteria *in the etiology of Crohn's disease (CD) has been a contentious subject for many years. Recently, our laboratory showed that spontaneous colitis in IL-10^-/- ^mice is driven in part by antigens (Ags) conserved in *Mycobacteria*. The present study dissects some of the common cellular and molecular mechanism that drive *Mycobacteria*-mediated and spontaneous colitis in IL-10^-/- ^mice.

**Results:**

We show that serum from inflammatory bowel disease (IBD) patients contain significantly higher levels of *Mycobacterium avium paratuberculosis*-specific IgG1 and IgG2 antibodies (Abs), serum amyloid A (SAA) as well as CXCR3 ligands than serum from healthy donors. To study the cellular mechanisms of *Mycobacteria*-associated colitis, pathogen-free IL-10^-/- ^mice were given heat-killed or live *M. avium paratuberculosis*. The numbers of mucosal T cells, neutrophils, NK/NKT cells that expressed TNFα, IFN-γ, and/or CXCL10 were significantly higher in mice that received live *Mycobacteria *than other groups. The numbers of mucosal CXCR3^+^, CXCL9^+^, CXCL11^+ ^and/or IFN-γ^+ ^dendritic cells (DCs) were also significantly higher in *M. avium paratuberculosis*-challenged mice, than compared to control mice.

**Conclusion:**

The present study shows that CD and UC patients mount significant *Mycobacteria*-specific IgG1 > IgG2 and CXCR3 ligand responses. Several cellular mechanisms that drive spontaneous colitis also mediate *Mycobacteria*-enhanced colitis in IL-10^-/- ^mice. Similar to IL-10^-/- ^mice under conventional housing, we show that *Mycobacteria*-challenge IL-10^-/- ^mice housed under otherwise pathogen-free conditions develop colitis that is driven by CXCR3- and CXCR3 ligand-expressing leukocytes, which underscores another important hallmark and molecular mechanism of colitis. Together, the data show that *Mycobacteria*-dependent host responses, namely CXCL10^+ ^T cells and NK cells, assist in the recruitment and activation of CXCR3^+ ^and CXCL11^+ ^leukocytes to enhance colitis of susceptible hosts.

## Background

Although increasing evidence suggests that intestinal flora is involved in the pathogenesis of IBD, to date no specific bacterial pathogen has been identified as the cause of this disease. The role of *Mycobacteria *in the etiology of IBD has been a contentious subject for many years. The possible association between *M. avium paratuberculosis *and CD in humans was first suggested nearly 100 years ago because Johne's disease (JD) and CD have similar histological and pathological features with affected tissues containing granulomas [1]. Interest in a causal association between JD and CD was stimulated in the 1980s after *M. avium paratuberculosis *was isolated from material taken from patients with CD [1,2]; subsequent studies confirmed these results [3,4]. This premise is further supported by successful CD treatment with anti-mycobacterials containing macrolide antibiotics [5] and from the sero-reactivity of the CD patient serum samples against *M. avium paratuberculosis *[6]. Recently, we reported that humoral and cellular responses during spontaneous colitis in IL-10^-/- ^mice are driven in part by conserved Ags in *Mycobacterium *species [7]. Together, these studies form a wide body of evidence that supports the potential role for *Mycobacteria *in CD. However, an incomplete understanding of the cellular mechanisms responsible for the pathology of colitis makes this conclusion uncertain.

There is a consensus that the mucosa of CD patients is dominated by Th1 cell producing inflammatory cytokines [8,9]. CXCR3 interactions with CXCL9, CXCL10 and CXCL11 are important for the selective homing of Th1 effectors cells [10], which mediate mucosal immunity, inflammation, and colitis [11,12]. IL-10^-/- ^mice develop spontaneous colitis that has similarities to human CD at ~3 months of age under conventional housing conditions which can be abrogated by anti-CXCL10 Ab treatment. The present study explores the CXCR3 axis and cellular mechanisms that drive *Mycobacteria*-associated colitis in IL-10^-/- ^mice. We also report that serum from both CD and UC patients contain significantly higher *Mycobacteria*-specific IgG1 and IgG2 Ab responses as well as CXCL9, CXCL10, CXCL11 and SAA levels than compared to normal healthy donors. These innate and adaptive immune responses coincide with *Mycobacteria*-specific humoral and cellular responses and modulation of mesenteric lymph node (MLN), Peyer's patch (PP) and lamina propria (LP) Th1-associated cytokines and CXCR3 ligands expressed by DCs, T cells, neutrophils and NK/NKT cell subsets following *Mycobacteria *challenge of otherwise specific pathogen-free mice.

## Methods

### Immunogens

*M. avium paratuberculosis *strain Ben (CIP 103966), a clinical isolate from a CD patient, was obtained from the American Type Culture Collection (ATCC# 43544). Bacteria were cultured in Middlebrook 7H9 broth supplemented with 10% ADC (BD/Difco) and 2 μg/ml mycobactin J (Allied Monitor) to an OD_580 _of 0.5 and frozen. The viable titer of these stocks was determined by thawing replicates, serial dilution in culture medium and plating on Middlebrook 7H10 agar supplemented with 2 μg/ml of mycobactin J. For detection of *Mycobacteria*-specific responses, *M. avium paratuberculosis *bacilli were fixed with 2% paraformaldehyde.

### Sera collection

Sera from 62 CD and 88 UC female patients and 32 normal healthy female donors were collected by Clinomics Biosciences, Inc. The ages of the patients were 20 to 41 years. Diagnoses were based on clinical, radiological, endoscopic, and histological criteria. These patients did not receive any steroid treatment before blood was drawn. All subjects gave written informed consent and the study was approved by the Clinomics Biosciences Research Ethics Committee, (Pittsfield, MA, USA). Subsequently, the University of Louisville Institutional Review Board (IRB) approved the use of these diagnostic specimens in accordance with the Department of Health and Human Service Policy for the Protection of Human Research Subjects 45 CFR 46.101(b) 2 and use of archived de-identified materials.

### *Mycobacterium*-specific Ab detection by ELISA

*Mycobacteria*-specific IgG Ab responses in the serum of human patients were measured by ELISA, according to manufacturer's protocol (Invitrogen-Zymed). Briefly, 96-well Falcon ELISA plates (Fisher Scientific) were coated with 100 μl of 1 μg/ml of heat-killed and paraformaldehyde-fixed *M. avium paratuberculosis *in coating buffer (sodium carbonate-bicarbonate buffer) or serially diluted human IgG1, IgG2, IgG3, and IgG4 isotype controls overnight at 4°C and blocked with 200 μl of 10% FBS (Atlanta Biologicals) in PBS (FBS-PBS) for 2 hours at room temperature. Individual samples were added and serially diluted in FBS-PBS. After 4 hours incubation at room temperature, the plates were washed (3 times), and the concentrations of IgG subclass Abs were determined by the addition 100 μl of biotin-conjugated mouse anti-human γ1 (Clone 8c/6-39 at 12.5 ng/ml), γ2 (Clone HP-6014 at 125 ng/ml), γ3 (Clone HP-6050 at 12.5 ng/ml), and γ4 (Clone HP-6025 at 50 ng/ml) heavy-chain-specific monoclonal Abs. After the incubation and washing steps, 100 μl of 0.5 μg/ml horse radish peroxidase (HRP)-conjugated anti-biotin Ab (Vector Labs) in FBS-PBS was added to IgG subclass Ab detection wells, and the plates were incubated for 2 hours at room temperature. Following incubation, the plates were washed 6 times and developed by adding 100 μl of ABTS solution and read at 415 nm. The *Mycobacterium*-specific ELISA assays were capable of detecting 8 pg/ml for human IgG subclass samples.

### Chemokine analysis by ELISA

Serum concentrations of human CXCL9, CXCL10 and CXCL11 were determined by ELISA (R&D Systems), according to the manufacturer's instructions. In brief, 96-well ELISA plates were coated with capture Abs, and the plates were incubated overnight at room temperature. After blocking, 100 μl of sample or standards were added to each well, and the plates were incubated for 2 hours at room temperature. Then, 100 μl of detection Abs solution was added to each well, and the plates were further incubated for 2 hours at room temperature. After washing the plates, 100 μl of streptavidin-HRP solution was added, and the plates were incubated for 20 min in the dark. Next, 100 μl of substrate solution was added to the plates, which were incubated for an additional 20 min at room temperature in the dark. Finally, 50 μl of the stop solution was added, and the plates were read at an optical density of 450 nm after 30 min using λ corrections of 540 and 570 nm. The ELISA assays were capable of detecting >10 pg/ml of each chemokine.

### SAA ELISA

SAA levels were determined by ELISA kit (Biosource International, Camarillo, CA). In brief, 50 μl of SAA-specific mAb solution was used to coat micro-titer strips to capture SAA. Serum samples and standards were added to wells and incubated for 2 hr at RT. After washing in the assay buffer, the HRP-conjugated anti-SAA mAb solution was added, and the plates were incubated for 1 hr at 37°C. After washing, 100 μl tetra-methyl-benzidine (TMB) substrate solution was added, and the reaction was stopped after incubation for 15 minute at RT. After the addition of stop solution, the plates were read at an optical density of 450 nm.

### Animals and *M. avium paratuberculosis *challenge

Female 4 to 5 weeks old IL-10^-/- ^mice, on B6 background, were purchased from Jackson Laboratories (Bar Harbor, ME) and used to establish a colony at the Morehouse School of Medicine animal facility. The animals were housed and maintained in isolator cages under specific-pathogen-free housing conditions. The guidelines proposed by the Committee for the Care of Laboratory Animal Resources Commission of Life Sciences-National Research Council were followed to minimize animal pain and distress. To determine the cellular responses enhanced by *Mycobacteria*, groups of naïve IL-10^-/- ^mice received 200 μl of 10^4 ^CFU live or heat-killed (pasteurized; 65°C for 2 hours) *M. avium paratuberculosis *in cream milk or cream milk alone (control) by oral gavage. Mice were sacrificed by CO_2 _inhalation 14 weeks post inoculation. Each experimental group consisted of 5 to 10 mice.

### Cell isolation

Spleens and MLN from individual mice were mechanically dissociated, and red blood cells were lysed with ACK lysis buffer (Sigma), 14 weeks after challenge. Single cell suspensions of the spleens and MLN were passed through a sterile wire screen (Sigma). Cell suspensions were washed twice in RPMI 1640 and stored on ice in complete medium containing 10% FBS until use. Cells from the LP and PP were isolated as described previously [13]. After removal, PP were kept on ice-cold RPMI containing 2% FBS. Intestines were cut into 1 cm stripes and stirred in PBS containing 1 mM EDTA at 37°C for 30 min. In brief, the intestinal LP was isolated by digesting intestinal tissue in collagenase type IV (Sigma) in RPMI 1640 (collagenase solution), for 45 min with stirring at 37°C. After each 45-minute interval, the released cells were centrifuged and stored in complete medium, and remaining mucosal pieces were replaced with fresh collagenase solution. LP lymphocytes were further purified using a discontinuous percoll (Pharmacia, Uppsala, Sweden) gradient, collecting between the 40/75% percoll interface. Leukocytes were maintained in a complete medium, which consisted of RPMI 1640 supplemented with 10 ml/L of nonessential amino acids (Mediatech, Washington, DC), 1 mM sodium pyruvate (Sigma), 10 mM HEPES (Mediatech), 100 U/ml penicillin, 100 μg/ml streptomycin, 40 μg/ml gentamycin (Elkins-Sinn, Inc., Cherry Hill, NJ), 50 μM mercaptoethanol (Sigma), and 10% FBS (Atlanta Biologicals).

### Flow cytometry analysis

Leukocytes from the spleen, MLN, PP, and LP were isolated as described above and stained to measure *ex vivo *expression of chemokines and cytokines, 14 weeks after challenge. Cells were first treated with Fc block (BD PharMingen, San Diego CA) for 15 min at 4°C, washed with staining buffer, and then stained with CY-, FITC- or PE-conjugated anti-CD3 (145-2C11), -CD4 (H129.19), -CD8 (LY-2 53-6.7), -CD11b (M1/70), -CD11c (HL3), and -NK1.1 (PK136) (BD-PharMingen, San Diego CA) for 30 min with shaking. The cells were washed 2 times with staining buffer and resuspended with BD Cytofix/Cytoperm (BD-PharMingen) solution for 10 min each. The cells were again washed 2 times in BD perm/wash solution at 4°C for 10 min each. The fixed, permeabilized cells were then resuspended in a pre-determined APC-conjugated anti-CXCL10, CXCL9, CXCL11, TNF-α, IFN-γ, or CXCR3 Ab for 30 min at 4°C in the dark. Lymphocytes were then washed with FACS staining buffer (PBS with 1% BSA) and fixed in 2% paraformaldehyde and analyzed by flow cytometry (Becton Dickinson, San Diego, CA).

### Histology

Intestinal tissues were preserved using Streck™ fixative (Streck Laboratories, LaVista, NE) and embedded in paraffin. Fixed tissues were sectioned at 6 μm and stained with hematoxylin and eosin for microscopic examination. Intestinal lesions were multi-focal and of variable severity. The grades given to intestinal sections that took into account the number of lesions as well as their severity. A score (0 to 4) was given, based on the criteria described previously [11]. The summation of these scores provided a total colonic disease score per mouse. The disease score could range from 0 to a maximum of 12 (with Grade 4 lesions in ascending, transverse, and descending colon segments).

### Statistics

The data expressed as the mean ± SEM and compared using a two-tailed paired student's *t*-test or an unpaired Mann Whitney *U*-test. The results were analyzed using the Statview II statistical program (Abacus Concepts, Inc., Berkeley, CA) and Microsoft Excel (Microsoft, Seattle, WA) for Macintosh computers. Single-factor and two-factor variance ANOVA analyses were used to evaluate groups and subgroups, respectively. Hence, results were considered statistically significant if *p *values were < 0.01.

## Results

### Changes in IgG subclass Ab profile in IBD patients

**Figure 1:**
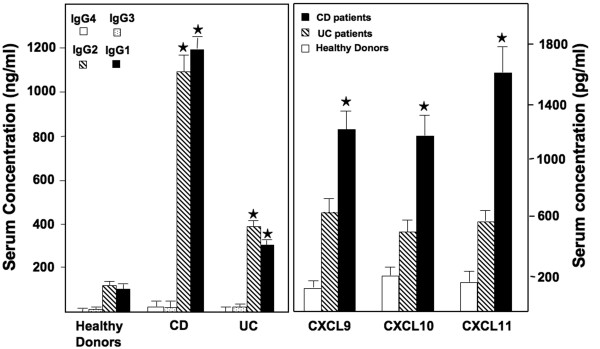
**Serum CXCR3 ligands and mycobacterial -specific Ab responses in IBD patients**. Sera from 62 CD and 88 UC female patients and 32 normal healthy female donors, not undergoing any treatment, were isolated and evaluated for the presence of CXCR3 ligands (i.e., CXCL9, CXCL10, and CXCL11) and mycobacterial-specific IgG1, IgG2, IgG3 and IgG4 Abs. These levels were determined by ELISAs capable of detecting 10 > pg/ml of these ligands. The data presented are concentrations ± SEM. Asterisk(s) indicate statistically significant differences, i.e., *p *< 0.01, between healthy donors and IBD patients.

Previously, we have shown that serum *Mycobacteria*-specific IgG2a Ab responses were higher in IL-10^-/- ^mice with spontaneous colitis, than compared to control mice [7]. This previously described imbalance of the Th1 > Th2 cytokine levels as well as humoral response (IgG2a > > IgG1) suggested that a Th1-biased host response is associated with the progression of colitis. To further test this hypothesis in human subjects, we measured levels of *Mycobacteria*-specific IgG subclass Abs in the sera of IBD patients and healthy donors. While total IgG1, IgG2, IgG3, and IgG4 subclass Abs were significantly higher in the sera of IBD patients compared to healthy donors (data not shown), the profile of the IgG humoral response in IBD patients also revealed increases in *Mycobacteria*-specific IgG1 and IgG2 Abs (Figure 1). These responses in CD patients were significantly higher than in UC patients or normal healthy donors. CXCR3 ligands were also increased in these samples than compared to healthy donors. These results suggest that IBD patients have higher CXCL9, CXCL10, and CXCL11 levels and *Mycobacteria*-specific IgG1 and IgG2 Ab responses. These findings correlate well with our previous findings showing higher levels of *Mycobacteria*-specific IgG2a and CXCR3 ligands during spontaneous colitis in IL-10^-/- ^mice under conventional housing [7].

### Characteristics of colitis progression

**Figure 2:**
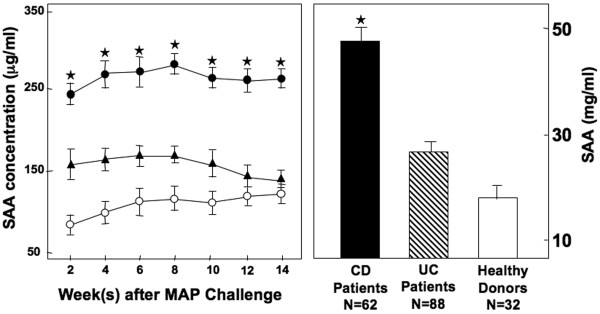
**Change in SAA levels in IBD patients and in IL-10^-/- ^mice after mycobacterial challenge**. Sera from 62 CD and 88 UC female patients and 32 normal healthy female donors, not undergoing any treatment, were isolated and evaluated for the presence of SAA. IL-10^-/- ^mice on B6 background, received 200 μl of cream milk alone (○; control) or cream milk containing 10^4 ^CFU of live (●) or heat-killed (▲) *M. avium paratuberculosis*. SAA levels during *Mycobacteria*-enhanced colitis as well as IBD patients and healthy donors were measured by ELISA. Experimental groups consisted of 5 mice, and experiments were repeated 3 times. The data presented are the mean ± SEM concentration of SAA. Asterisks indicate statistically significant differences, i.e., *p *< 0.01, between control and *Mycobacteria*-treated groups or healthy donors and IBD patients.

**Figure 3:**
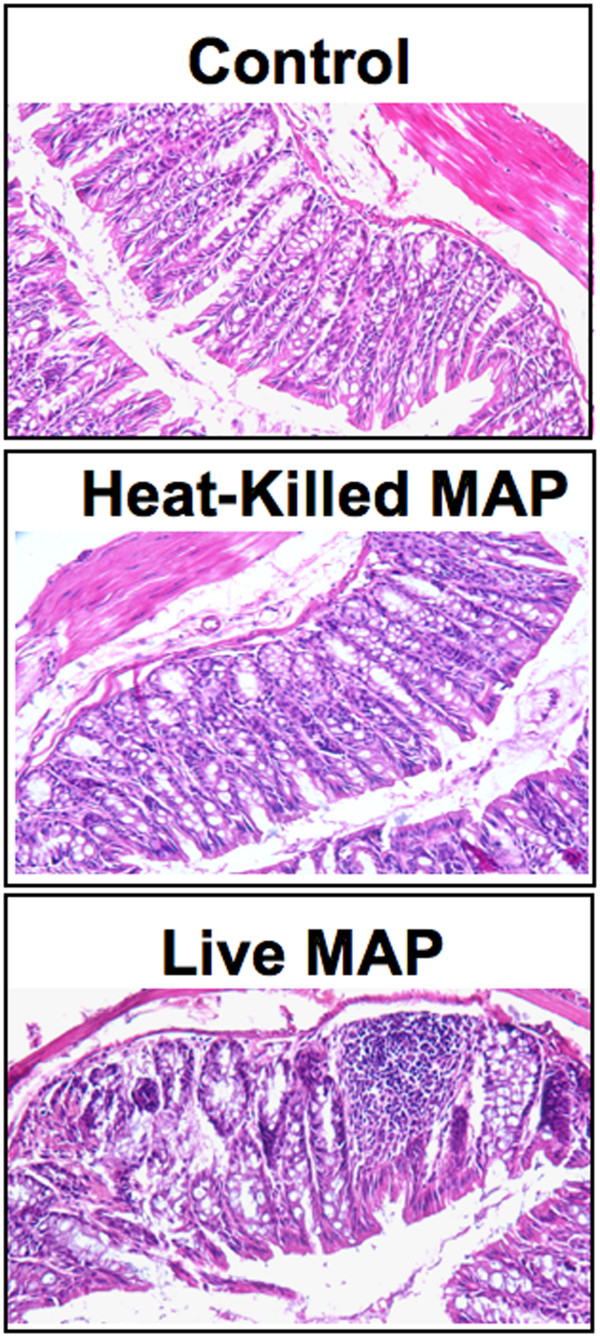
**Intestinal histological characteristics of IL-10^-/- ^mice challenged with *Mycobacteria***. IL-10^-/- ^mice on B6 background, received 200 μl of cream milk alone (○; control) or cream milk containing 10^4 ^CFU of live (●) or heat-killed (▲) *M. avium paratuberculosis*. After sacrifice, intestines were fixed, sectioned at 6 μm, and stained with hematoxylin and eosin. Sections were examined by light microscopy. Experimental groups consisted of 5 mice and experiments were repeated 3 times.

We also measured the SAA levels of IBD patients and healthy donors; CD patients displayed significantly higher SAA levels compared to other groups, which correlated with the higher *Mycobacteria*-specific IgG2a levels described above. Colitis progression in IL-10^-/- ^mice closely corresponds with increased SAA levels (≥ 250 μg/ml) and with reduction in body weight, compared with the initial body weight [9,11,12]. The results of the present study show that mice challenged with live *Mycobacteria *in otherwise specific pathogen-free conditions experienced a significant rise in SAA levels when compared to similar mice challenged with heat-killed *Mycobacteria *or control mice (Figure 2). Despite the significant increase in SAA and more pronounced reduction in body mass, both live and heat-killed *Mycobateria *challenged IL-10^-/- ^mice developed histologically similar colitis than compared to controls, as illustrated by significantly higher colitis disease scores (Table 1). The intestinal tissues of mice challenged with *Mycobacteria *showed higher increases in leukocyte infiltrates, which consisted of lymphocytes and occasionally polymorphonuclear cells as well as a higher frequency of lymphoid follicles in live *versus *heat-killed *Mycobacteria*-challenged groups (Figure 3). In short, colitis was more aggressive in mice that received live *Mycobacteria*, as noted by multi-focal lesions and aggregates of leukocyte infiltrates in the large intestines, than compared to control mice.

**Table 1:** Histological evaluation of *Mycobacteria*-enhanced colitis in IL-10^-/- ^mice

Treatment/Group	Number of Mice	Colitis Disease Score (0 – 12)
Live *M. avium paratuberculosis*	10	6.12 ± 2.01*
Pasteurized *M. avium paratuberculosis*	10	4.89 ± 1.41*
Control (vehicle only)	10	1.45 ± 0.79
Untreated wild-type B6	5	0

The severity of colitis was monitored by evaluating the histopathological changes in colons of IL-10^-/- ^mice on B6 background that received 200 μl of cream milk or 10^4 ^CFU of live or heat-killed *M. avium paratuberculosis *in cream milk. Following the sacrifice of mice 14 weeks later, colons were fixed, sectioned at 6 μm and stained with hematoxylin and eosin. The sections were microscopically examined and scored for the severity of colitis. The data presented are the mean ± SEM colitis score. Asterisks indicate statistically significant differences, i.e., *p *< 0.01 between experimental and the control group.

### CXCR3, CXCL11, IFN-γ, and/or TNF-α expression by CD4^+ ^and CD8^+ ^T cells

**Figure 4:**
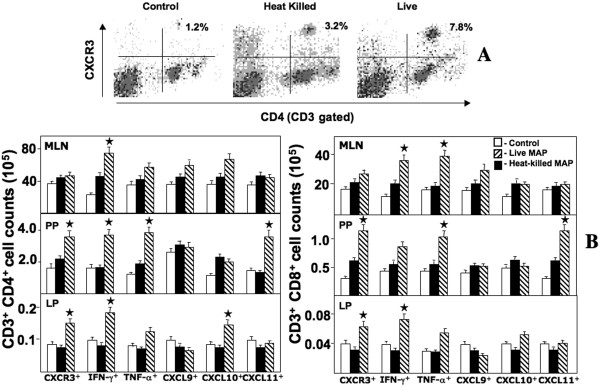
**Changes in the T cell populations expressing CXCR3, IFN-γ, TNF-α, and CXCR3 ligands in IL-10^-/- ^mice after mycobacterial challenge**. MLN, PP, and LP lymphocytes were isolated from IL-10^-/- ^mice challenged with cream milk alone (control) or 10^4 ^CFU of heat-killed or live *M. avium paratuberculosis *in cream milk. Changes in the frequency of LP CXCR3^+ ^CD4^+ ^T cells from heat-killed or live *Mycobacteria*-challenged and control mice (Panel A). Changes in the number (± SEM) of total IFN-γ-, TNF-α-, CXCR3-, CXCL9-, CXCL10- or CXCL11-expressing CD4^+ ^and CD8^+ ^T cell lymphocytes from control mice as well as heat-killed or live *Mycobacteria*-challenged IL-10^-/- ^mice are shown in Panel B. Asterisks indicate statistically significant differences, i.e., *p *< 0.01, between control and *Mycobacteria*-treated groups.

To address the leukocyte subsets responsible for colitis progression, we assessed the *ex vivo *expression of chemokines and cytokines by MLN-, PP-, and LP-derived cell subsets in IL-10^-/- ^mice challenged with live or heat-killed *M. avium paratuberculosis *bacilli. The numbers of CD4^+ ^and CD8^+ ^T cells from MLN, PP, and LP lymphocytes that expressed CXCR3, IFN-γ, and TNF-α from mice challenged with live bacilli were significantly higher than similar mice challenged with heat-killed bacilli or the control group. Dramatic increases in the number of PP CXCL11^+ ^T cells, PP and LP CXCR3^+ ^T cells and PP TNF-α^+ ^T cells as well as MLN TNF-α^+ ^CD8^+ ^T cell subsets were observed in mice challenged with live bacilli than compared to other groups (Figure 4). PP, MLN, and LP IFN-γ^+ ^CD4^+ ^T cells as well as MLN and LP IFN-γ^+ ^CD8^+ ^T cells were significantly elevated after livemycobacterial challenge. These results imply that intestinal inflammation is driven in part by effector site-derived (PP and LP) CXCR3^+ ^TNF-α^+ ^CD4^+ ^and CD8^+ ^T cells along with similar cells from PP that produce CXCR3 ligands as well as MLN (inductive site) IFN-γ^+ ^T cells to presumably support Th1-biased colitis.

### Alteration of DC cytokine and CXCR3 ligands expression following *Mycobacteria *challenge

**Figure 5:**
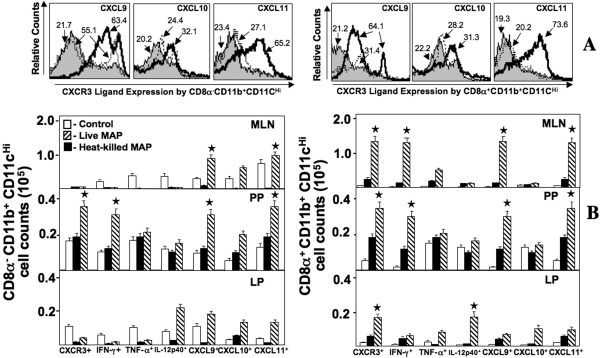
**Changes in CD8α ^+ ^and CD8α ^- ^CD11b^+^CD11c^+ ^cells expressing CXCR3, IFN-γ, TNF-α, IL-12p40, and CXCR3 ligands in IL-10^-/- ^mice after mycobacterial challenge**. MLN, PP, and LP lymphocytes were isolated from IL-10^-/- ^mice challenged with cream milk alone (control) or cream milk containing 10^4 ^CFU of live or heat-killed *M. avium paratuberculosis*. Changes in the mean fluorescent intensities of CXCL9, CXCL10, and CXCL11 expressed by MLN CD8α^+ ^and CD8α^- ^CD11b^+^CD11c^+ ^cells from control mice (shaded histogram) and heat-killed (dotted outlined histogram)- or live *Mycobacteria *(solid outlined histogram)-challenged mice are shown in Panel A. Changes in the number (± SEM) of CXCR3-, IFN-γ-, TNF-α-, IL-12p40-, CXCL9-, CXCL10- or CXCL11-expressing CD8α^+ ^and CD8α^- ^CD11b^+^CD11c^+ ^cells from control mice as well as live or heat-killed *Mycobacteria*-challenged IL-10^-/- ^mice are shown in Panel B. Asterisks indicate statistically significant differences, i.e., *p *< 0.01, between control and *Mycobacteria*-treated groups.

To further address the adaptive *versus *innate leukocyte subsets responsible for *Mycobacteria*-enhanced colitis in IL-10^-/- ^mice, we next assessed myeloid and plasmacytoid DC phenotypes from MLN, PP, and LP after disease onset. PP and MLN DCs expressed elevated levels of CXCL9 and CXCL11, but not CXCL10, during *Mycobacteria*-enhanced colitis as indicated by their relative mean fluorescent intensities (Figure 5A). Interestingly, both DC subsets heterogeneously expressed CXCL9. The number of PP CXCR3^+ ^DCs was significantly higher in live *Mycobacteria*-challenged mice than in other groups (Figure 5B). However, the number of MLN CXCR3^+ ^plasmacytoid DCs was significantly higher in mycobacterial-challenged mice than in other groups of mice. The numbers of MLN and LP DCs subsets expressing CXCL9 and CXCL11 after the onset of colitis were significantly increased during live *Mycobacteria*-enhanced colitis than in the other groups with the exception of MLN CXCL10^+ ^myeloid DCs that were elevated following live bacterial challenge. While the DCs subsets studied did not appear to significantly express TNF-α, there were significantly more numbers of MLN and PP CD8α^+ ^DCs and only PP-derived CD8α^- ^DCs that expressed IFN-γ from mice with live *Mycobacteria*-enhanced colitis, than compared to other groups. LP plasmacytoid DCs from mice receiving live bacteria were also increasingly IL-12p40^+^, than compared to other groups.

### CXCR3, IFN-γ, TNF-α, IL-12, CXCL9, CXCL10 and CXCL11 expression by NK and NKT cells during *Mycobacteria*-enhanced colitis

**Figure 6:**
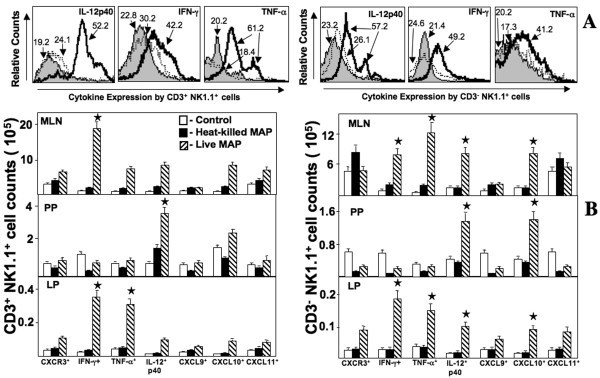
**Changes in NK and NKT cells of IL-10^-/- ^mice challenged with *Mycobacteria***. MLN, PP, and LP lymphocytes were isolated from IL-10^-/- ^mice challenged with cream milk alone (control) or cream milk containing 10^4 ^CFU of live or heat-killed *M. avium paratuberculosis*. Changes in the mean fluorescent intensities of IL-12p40, IFN-γ, and TNF-α expressed by LP CD3^+ ^and CD3^- ^NK1.1^+ ^cells from control mice (shaded histogram) or heat-killed (dotted outlined histogram)- and live MAP (solid outlined histogram)-challenged mice are shown in Panel A. Changes in the number (± SEM) of CXCR3-, IFN-γ-, TNF-α-, IL-12p40-, CXCL9-, CXCL10- or CXCL11-expressing by CD3^+ ^and CD3^- ^NK1.1^+ ^cells from control mice and live or heat-killed *Mycobacteria*-challenged IL-10^-/- ^mice are shown in Panel B. Asterisks indicate statistically significant differences, i.e., *p *< 0.01, between control and *Mycobacteria*-treated groups.

NK cells act as regulators of CD4^+ ^T cell-driven colitis [14]. While neither NK nor NKT cell subsets largely expressed CXCR3, there were substantially more CXCL10^+ ^NK cells in the MLN, PP, and LP during *Mycobacteria*-enhanced colitis, than compared to other groups (Figure 6). IL-12p40 expression and the number of NK cell subsets were elevated in mice challenged with live *Mycobacteria *than compared to mice challenged with heat-killed *Mycobacteria *or control mice. Whereas, greater numbers of IFN-γ^+ ^NK and NKT cells were confined to the MLN and LP in live *Mycobacteria*-challenged group than compared to other groups. In contrast, we noticed a modest decrease in the number of PP CXCR3^+^, IFN-γ^+^, CXCL9^+ ^and CXCL11^+ ^NK cells during live *Mycobacteria*-enhanced colitis. These results suggest that together NK and NKT cells express CXCL10, IL-12p40, TNF-α and IFN-γ to recruit and activate CXCR3^+ ^leukocytes to inductive and effector sites during *Mycobacteria*-enhanced colitis.

### Th1-associated cytokines, CXCR3 and CXCR3 ligands expressed by neutrophils

**Figure 7:**
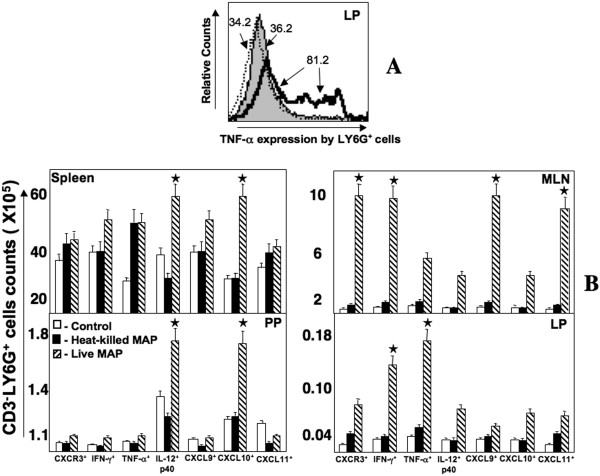
**Changes in cell populations of neutrophils during *Mycobacteria*-enhanced colitis**. Spleen, MLN, PP, and LP lymphocytes were isolated from IL-10^-/- ^mice challenged with cream milk alone (control) or cream milk containing 10^4 ^CFU of live or heat-killed *M. avium paratuberculosis*. Changes in the mean fluorescent intensities of IL-12p40, IFN-γ, and TNF-α expressed by LP CD3^- ^LY6G^+ ^cells from control mice (shaded histogram) or heat-killed (dotted outlined histogram)- and live *Mycobacteria *(solid outlined histogram)-challenged mice are shown in Panel A. Changes in the number (± SEM) of CXCR3-, IFN-γ-, TNF-α-, IL-12p40-, CXCL9-, CXCL10- or CXCL11-expressed by CD3^- ^LY6G^+ ^cells from control mice and live or heat-killed *Mycobacteria*-challenged IL-10^-/- ^mice are shown in Panel B. Asterisks indicate statistically significant differences, i.e., *p *< 0.01, between control and *Mycobacteria*-treated groups.

Neutrophils are among the first cells to arrive at sites of inflammation and infection. These cells also have the capacity to release CXCL10 [15] and are present during *Mycobacteria*-enhanced colitis. LP neutrophils significantly expressed TNF-α, in a heterogenous fashion, following live mycobacterial challenge (Figure 7A). TNF-α^+ ^neutrophils were largely retained in the LP during disease when compared to other groups. MLN and LP, but not PP or spleen, contained higher numbers of TNF-α^+ ^and IFN-γ^+ ^neutrophils during *Mycobacteria*-enhanced colitis (Figure 7B). In contrast, higher numbers of IL-12p40^+ ^and CXCL10^+ ^LY6G^+ ^cells were found in PP and spleen, but not in MLN of LP from live *Mycobacteria*-challenged mice relative to control mice and heat-killed *Mycobacteria*-challenged mice. However, greater numbers of CXCL9^+ ^and CXCL11^+ ^neutrophils were found in MLNs of live bacteria-challenged mice, than compared to other groups. These data suggest that neutrophils are compartmentalized and tightly regulated to differentially express CXCR3 ligands, CXCR3 and Th1-promoting cytokines during *Mycobacteria*-driven colitis.

## Discussion

Although there is no recognized or agreed on infectious agent for IBD, interest in *M. avium paratuberculosis *as a possible causative began due to similar signs and symptoms of JD and CD. For example, chronic granulomatous and intramural inflammation is common in both CD and JD; however, the precise pathophysiologic mechanisms sustaining inflammation in these diseases are imprecisely known. In confirmation with others [16,17], we show an increase in *Mycobacteria*-specific IgG1 and IgG2 Abs in sera from patients with active CD or UC. While the cellular mechanism(s) and response(s) mediating *Mycobacteria*-enhanced IBD have not been entirely demonstrated, recently we showed that colitis in IL-10^-/- ^mice was driven in part by 85B Ag-specific CD4^+ ^T cell responses [7]. As *M. avium paratuberculosis *is a mycobactin auxotroph and is not known to replicate outside a host, spontaneous colitis of IL-10^-/- ^mice upon transfer to conventional housing is possibly the result of exposure to *Mycobacterium *species, which are commonly found in soil and water. We illustrate several cellular mechanisms that may drive *Mycobacteria*-enhanced colitis in IL-10^-/- ^mice. *Mycobacteria*-challenged IL-10^-/- ^mice housed under otherwise pathogen-free conditions develop colitis that is driven by CXCR3- and corresponding ligand(s)-expressing leukocytes, which underscores another important hallmark or molecule mechanism of colitis.

The expression of CXCR3 and its ligands (CXCL10 >> CXCL11 or CXCL9) by mucosal T cells significantly increased in mice challenged with live *Mycobacteria *than compared with similar mice challenged with heat-killed *Mycobacteria *or controls. Our results also suggest plasmacytoid > > myeloid DCs express CXCL9 and CXCL11, but less CXCL10 as well as IFN-γ to recruit, differentiate, and expand Th1 cells to mediate colitis. NK cell and neutrophil subsets were compartmentalized in mucosal inductive and/or effector site to further foster adaptive Th1 responses through IFN-γ and IL-12p40 production as well as TNF-α expression during colitis. Relatively high levels of serum TNF-α and IFN-γ as well as Ag-specific Th1 responses were also noted in this model [7]. These findings suggest an association between *Mycobacteria *and CD patients as well as IL-10^-/- ^murine colitis.

It is well established that IL-12 drives Th1 differentiation and subsequent IFN-γ production [18]. We demonstrate that local and/or systemic expression of the TNF-α, IFN-γ, and CXCR3 ligands are increased during live *Mycobacteria*-enhanced colitis. TNF-α is a proinflammatory cytokine produced by leukocytes at local sites of inflammation [19]. This cytokine is also important for the activation of granulocytes and fibroblasts during CD [20]. LP lymphocytes produce significant amounts of TNF-α in IBD tissues and secretary fluids [21-23]. Perhaps the production of CXCR3 ligands along with IFN-γ and TNF-α during colitis progression creates a situation in which Th1 cells are recruited, activated, further differentiated, and expanded to initiate or maintain disease. While TNF-α produced by CD4^+ ^T cells is neither sufficient nor required for the induction of murine colitis, its production by Ag-presenting cells is essential for the histopathological and clinical signs of colitis [24]. We show that NK cell subsets and neutrophils, compared to T cells, expressed the greatest levels of TNF-α during *Mycobacteria*-mediated colitis, which is also a hallmark of spontaneous murine colitis.

The presence of CXCL10 is considered an important signal for selective homing and/or activation of effector cells, which preferentially accumulate at inflammatory sites [25]. Previously, we showed CXCL10 enhanced CD28 and CD40 expression by Ag-activated CD4^+ ^T cells [12] and DC activation [26]. Plasmacytoid DCs have an inherent capacity to produce large amounts of IFN-γ and DC activation is a critical step and is reported to be higher in CD patients [27]. Intestinal LP DCs prime T cells and home to MLN for further activation and clonal expansion of T cells [28,29]. DCs can be found in the intestinal mucosal [30] and have also been shown to produce and respond to CXCR3 ligands [31]. In the present study, we show that CXCL9 and CXCL11 are predominantly produced by DCs in the MLN as well as in PP and to a lesser degree in the intestinal LP. The selective expression of CXCL9 and CXCL11 by plasmacytoid and myeloid DCs suggests a differential role for these cells in the attraction of specific lymphocyte subsets [10] as well as in the preferential recruitment and activation of Th1 cells [11,32]. In a recent study, mature DC-derived CXCR3 ligands were shown to be essential in Th1 cell retention in draining lymph nodes and to optimize Th1-mediated immune responses [33].

NK cells have been shown to be involved in the differentiation of naïve CD4^+ ^T cells into Th1 cells [34]. The current study suggests NK and NKT cells collaborate to produce CXCL10, IL-12p40, TNF-α, and IFN-γ to recruit and stimulate CXCR3^+ ^leukocytes in either inductive or effector sites during colitis. In IBD, neutrophil activation, migration, and degranulation are important effector mechanisms of intestinal damage [35]. It has been reported that human neutrophils have the capacity to release CXCL10 [15]. In the present study, changes in the number of neutrophils that expressed CXCR3, IFN-γ, CXCL9, and CXCL11 in the MLN, IL-12p40 and CXCL10 in PP as well as IFN-γ and TNF-α in LP of mice during *Mycobacteria*-enhanced colitis suggest that neutrophil subsets are differentially compartmentalized and regulated to propagate colitis.

Activation of toll-like receptors (TLRs) leads to the induction of antimicrobial pathways central to innate defense as well as upregulation of Ag presentation molecules and secretion of cytokines that influence the nature of the subsequent adaptive immune response. To this end, TLR signal transduction requires MyD88 [36,37]; MyD88^-/- ^mice are highly susceptible to infection by *M. tuberculosis *and colitis [38]. TLR-activated DCs generally favor Th1 responses due in large part to the production of IL-12 [39,40]. *Mycobacteria *cell constituents have long been recognized as powerful immunologic adjuvants. It is plausible that *Mycobacteria *stimulate TLRs to promote colitis in IL-10^-/- ^mice, which presents a scenario where mycobacterial recognition via TLR and MyD88 activate DCs to control Th1 polarization and expansion.

Live orally administered *M. avium paratuberculosis *do not readily colonize or extensively replicate in the murine host. The ability of this bacterial species to colonize and replicate in man is also questionable. In fact, B6 mice are only modestly susceptible to orally administered *M. avium paratuberculosis *[41]. Granulomatous lesions containing acid-fast bacteria develop in mesenteric lymph nodes 11 months after oral challenge using 10^9 ^CFU. In this study, 10^4 ^CFU of live *M. avium paratuberculosis *initiated the development of colitis, but there was no evidence of infection by histology (data not shown) or bacterial culture [7]. Presumably, IL-10 deficiency or a host that elicits Th1-biased immune responses to microbial challenge supports the development of colitis with either undetectable or no growth of *M. avium paratuberculosis*.

We speculate that innate (i.e., TLR agonists) and adaptive (e.g., 85B protein) immunity antigens are better presented by live *M. avium paratuberculosis *or recognized by the Th1-biased host to modulate relevant cellular and molecular mechanisms required by colitis development. Indeed, persistent presentation of flora present in conventional housing is required for colitis in IL-10^-/- ^B6 mice. Hence, heat-killed mycobacteria may not have allowed for this sustained presentation and expression in the host. Additional studies using antibiotics along with or without live or heat-killed bacteria (e.g., *Citrobacter rodenticum, Mycobacterium avium hominissuis*, etc.) will be necessary to reveal the precise requirements for *Mycobacteria*-induced colitis.

## Conclusion

**Figure 8:**
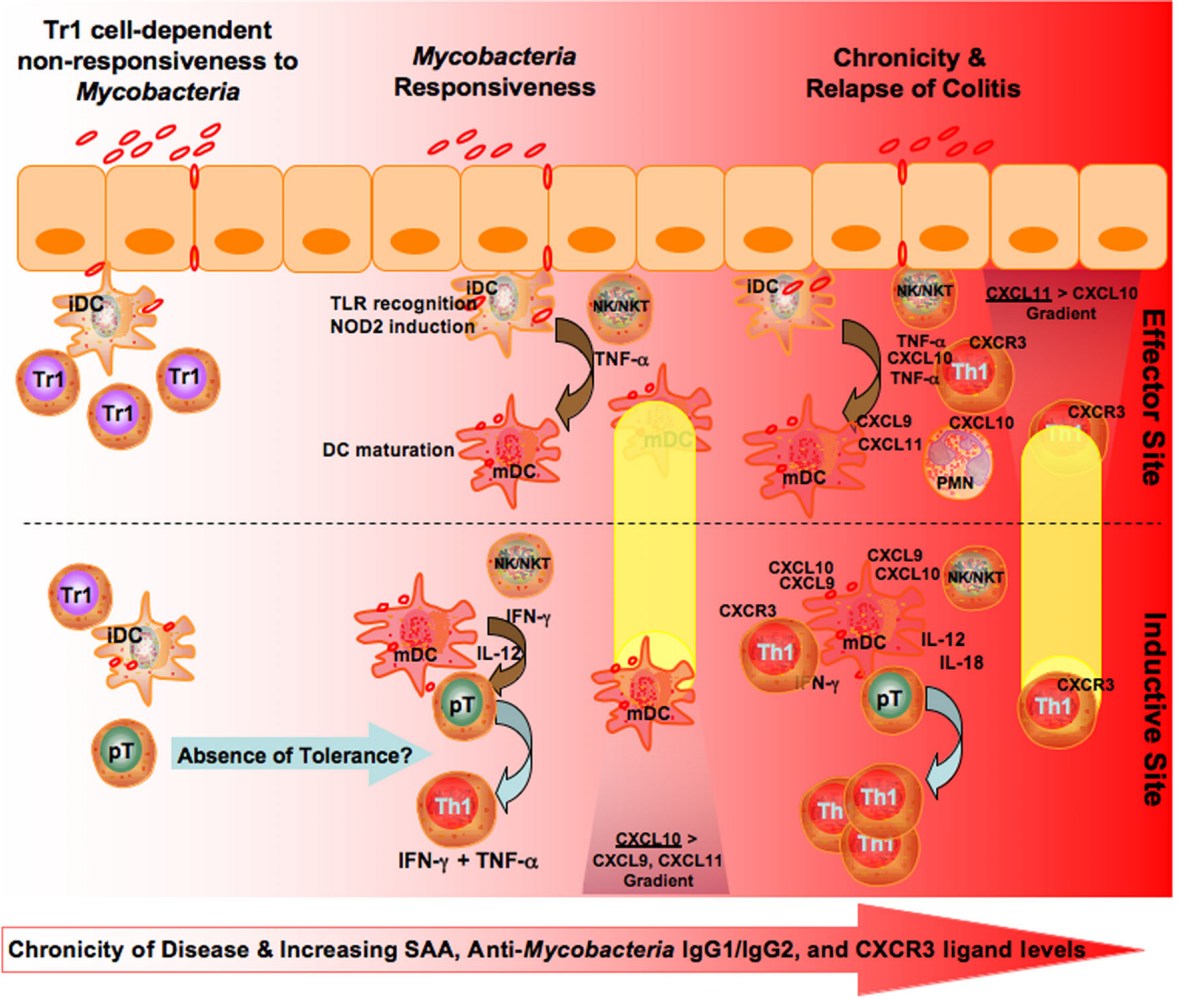
**Possible cellular and molecular mechanisms of *Mycobacteria*-mediated colitis**. The differential production of CXCR3 ligands and their recognition by CXCR3^+ ^cells are involved in *Mycobacteria*-enhanced colitis. Regulatory T cells (Tr1) are critical to maintain tolerance or homeostasis in the presence of commensal flora. In a host (e.g., IL-10^-/- ^or NOD2/CARD15 or TLR polymorphisms) that is deficient in the regulation of tolerance, inactive dendritic cells (iDC) mature and aid in the differentiation of precursor T helper cells (pT) to Th1 cells. These Th1 cells express CXCR3, TNF-α, and IFN-γ, while mature dendritic cells (mDC) and other activated antigen-presenting cells express CXCR3, CXCL9, CXCL11, and IL-12p40 to support Th1 development as well as the recruit of CXCR3- and CXCL10-expressing cells (e.g., CD8^+ ^T cells, polymorphonuclear cells (PMN), natural killer (NK) and NK T cells (NKT)) for the propagation and recurrence of IBD, which correlates with increases in SAA, CXCR3 ligands, and anti-*Mycobacteria *IgG1 and IgG2 Abs.

We can conclude that IBD patients have higher CXCL9, CXCL10, and CXCL11 levels and *Mycobacteria*-specific IgG1 and IgG2 Ab responses. These findings correlate with our previous findings that show higher levels of *Mycobacteria*-specific IgG2a and CXCR3 ligands occur during spontaneous colitis in IL-10^-/- ^mice [7]. In short, colitis was more aggressive in IL-10^-/- ^mice that received live *Mycobacteria*, than compared to similar mice that received heat-killed bacilli or vehicle, housed under specific pathogen-free housing. It can be surmised that IL-12p40^+ ^TNF-α^+ ^IFN-γ^+ ^NK and NKT cells express CXCL10 to recruit and activate CXCR3^+ ^leukocytes to inductive and effector sites during *Mycobacteria*-enhanced colitis (Figure 8). As with these innate immunity cells, neutrophils are compartmentalized in the MLN and tightly regulated to differentially express CXCL9 and CXCL11, CXCR3 and Th1-associated cytokines during *Mycobacteria*-mediated colitis. These results further show that murine colitis is driven in part by effector site-derived CXCR3^+ ^TNF-α^+ ^CD4^+ ^and CD8^+ ^T cells as well as inductive site IFN-γ^+ ^T cells to support Th1-biased colitis. This response is enhanced or maintained by high numbers of MLN and PP CD8α^+ ^IFN-γ^+ ^DCs, PP-derived CD8α^- ^IFN-γ^+ ^DCs, and IL-12p40^+ ^LP plasmacytoid DCs. While additional studies will be required to ascertain the precise role of *Mycobacteria *in the induction and progression of colitis, we report that live mycobacterial challenge accelerates chronic colitis in IL-10^-/- ^mice.

## Competing interests

The authors declare that they have no competing interests.

## Abbreviations

Ag, antigen; BrdU, 5-Bromo-2'-deoxy-uridine; CD, Crohn's disease; CFU, colony forming unit; CXCL9, MIG, monokine-induced by IFN-γ; CXCL10, IP-10, IFN-γ-inducible protein 10; CXCL11, I-TAC, IFN-γ-inducible T cell-α chemoattractant; DCs, dendritic cells; IBD, inflammatory bowel disease; JD, Johne's disease; LP, lamina propria; MLN, mesenteric lymph nodes; pDCs, plasmacytoid dendritic cells; PP, Peyer's patches; SAA, serum amyloid A; TLR, toll-like receptor; UC, ulcerative colitis.

## Authors' contributions

UPS carried-out all animal studies, quantified serum CXCR3 ligands, performed flow cytometry acquisition and analyzed data with the assistance of SS and RS. RKK and FDQ cultured *Mycobacterium avium paratuberculosis*, determined the corresponding CFU for challenge. DDT coordinated and performed the CXCR3 ligand and SAA serum ELISA of IBD patients. JWL conceived the study, participated in its design with all authors, coordinated and helped to draft the manuscript with the assistance of all authors. All authors read and approved the final manuscript.
